# Association of obstructive sleep apnea with hypertension: A systematic review and meta-analysis

**DOI:** 10.7189/jogh.08.010405

**Published:** 2018-06

**Authors:** Haifeng Hou, Yange Zhao, Wenqing Yu, Hualei Dong, Xiaotong Xue, Jian Ding, Weijia Xing, Wei Wang

**Affiliations:** 1 School of Public Health, Taishan Medical University, Taian, China; 2 School of Medical and Health Sciences, Edith Cowan University, Perth, Australia; 3 School of Basic Medical Science, Taishan Medical University, Taian, China; 4 Taishan Hospital of Shandong Province, Taian, China; * These authors contributed equally to the article

## Abstract

**Background:**

Obstructive sleep apnea (OSA) is a sleep disorder characterized as complete or partial upper airflow cessation during sleep. Although it has been widely accepted that OSA is a risk factor for the development of hypertension, the studies focusing on this topic revealed inconsistent results. We aimed to clarify the association between OSA and hypertension, including essential and medication-resistant hypertension.

**Methods:**

The Preferred Reporting Items for Systematic Reviews and Meta-Analyses (PRISMA) was followed. PubMed and Embase databases were used for searching the relevant studies published up to December 31, 2016. A quantitative approach of meta-analysis was performed to estimate the pooled odds ratio (OR) and 95% confidence interval (CI).

**Results:**

Twenty-six studies with 51 623 participants (28 314 men, 23 309 women; mean age 51.8 years) met inclusion criteria and were included in this study. Among them, six studies showed a significant association between OSA and resistant hypertension (pooled OR = 2.842, 95% CI = 1.703-3.980, *P* < 0.05). Meanwhile, the combination of 20 original studies on the association of OSA with essential hypertension also presented significant results with the pooled ORs of 1.184 (95% CI = 1.093-1.274, *P* < 0.05) for mild OSA, 1.316 (95% CI = 1.197-1.433, *P* < 0.05) for moderate OSA and 1.561 (95% CI = 1.287-1.835, *P* < 0.05) for severe OSA.

**Conclusions:**

Our findings indicated that OSA is related to an increased risk of resistant hypertension. Mild, moderate and severe OSA are associated essential hypertension, as well a dose-response manner relationship is manifested. The associations are relatively stronger among Caucasians and male OSA patients.

Obstructive sleep apnea (OSA) is a sleep disorder characterized as complete or partial upper airflow cessation during sleep, which mainly results from narrowed oropharyngeal or velopharyngeal anatomy [1,2]. OSA is reported to affect approximately 17% of American adults [3]. Hereditable dimension of craniofacial skeleton, obesity, and aging are considered risk factors for OSA, and the lack of neurophysiological regulation on airway dilator muscles also contributes to the causality of OSA [4]. Apart from causing uncomfortable symptomatology, untreated OSA is widely acknowledged to be associated with diabetes, cardiovascular disease and cerebrovascular disease [5]. Observational studies have illustrated that the prevalence of OSA is over 30% among hypertension patients and nearly 80% among resistant hypertensive patients [6,7]. Although the association between OSA and hypertension is considered obvious, the published results regarding this relationship are not consistent [3]. More than eight studies reported that OSA is not associated with hypertension, arousing skepticism of the effect of OSA on the risk for hypertension [2,8]. To our knowledge, the effect of continuous positive airway pressure (CPAP) therapy on blood pressure (BP) reduction improves the establishment of the causal association of OSA with hypertension and cardiovascular diseases [8]. However, a recent meta-analysis demonstrated that CPAP intervention does not reduce cardiovascular risk [9]. Besides another meta-analysis on CPAP treatment trials found a low reduction of HP (2.6 mm Hg for systolic BP and 2.0 mm Hg for diastolic BP) among OSA participants [10]. It has been manifested that CPAP may not be a sole hypertension intervention option for OSA patients [11]. To determine if OSA plays an independent causal role in hypertension, we conducted this systematic review and meta-analysis, and synthesized the studies on the association of OSA with essential hypertension and medication-resistant hypertension.

## MATERIALS AND METHODS

We followed the criteria of the Preferred Reporting Items for Systematic Reviews and Meta-Analyses (PRISMA). The reported PRISMA Checklist is provided in Table S1 in** Online Supplementary Document(Online Supplementary Document)**. The protocol of this study has been registered in PROSPERO (No. CRD42017064336), available at https://www.crd.york.ac.uk/PROSPERO/.

### Search strategy

The databases of PubMed and Embase were searched for literature published up to December 31, 2016. The search strategy was designed as a combination of the following key words: “obstructive sleep apnea” or “obstructive sleep apnea syndrome” or “sleep apnea” or “sleep disordered breathing” or “OSA” or “OSAS” or “SDB” and “hypertension” or “HTN”. In addition, studies cited in the references at the retrieved articles were further screened.

### Selection criteria and quality assessment

Two authors independently reviewed the title, abstract and full text of the publications to determine the suitability for inclusion. Eligible studies were included based on the following criteria: 1) studies conducted on human populations; 2) studies conducted to investigate the relationships between OSA and essential and/or resistant hypertension; 3) case-control studies, case-control comparison of baseline data in cohort populations or clinical populations, and cohort studies; 4) odds ratios (OR) and 95% confidence interval (CI) available or can be calculated; 5) OSA patients diagnosed with polysomnography, and classified with apnea-hypopnea index (AHI); 6) studies published in English. We evaluated the methodological quality of each study according to the quality assessment scale by reference to PRISMA statement and MOOSE guideline (Table S2 and Table S3 in **Online Supplementary Document(Online Supplementary Document)**).

### Data extraction

Original data were retrieved independently from the eligible publications by two authors. The following data were extracted: author’s name, year of publication, ethnicity of participants, type of study design, sample size, characteristics of the participants. If results derived from the same population were published more than once, the most recent publication or the one with the largest population size was enrolled. If a publication investigated the baseline data, and also studied the association of recent-onset hypertension with OSA among different numbers of participants through a follow-up, the investigations were treated as two independent studies. Data in all subgroups were collected when the authors reported results with age or gender stratification. The adjusted ORs were extracted from articles.

### Statistical analysis

All statistical analyses were performed with the Stata14.0 software (Stata Corp, College Station, TX, USA). The pooled ORs and 95% CIs were calculated to assess the strength of the association between OSA and hypertension. Q test and I^2^ statistic were used to evaluate heterogeneity across the involved studies. When I^2^<50% and *P* > 0.10, the fixed effect model was used to combine data sets. Otherwise, the random effect model was applied. Moreover, we conducted subgroup meta-analysis based on ethnicity of the population when heterogeneity was ultra (I^2^>50% or *P* < 0.10). To assess the robustness of the combined results and evaluate the effect of individual studies on this meta-analysis, we carried out sensitivity analysis by removing each study one at a time. Subsequently, the publication bias was assessed by the Funnel Plots analysis, and Egger’s regression asymmetry test was further complemented. The Trim and Fill method was used to adjust the significant publication bias.

## RESULTS

### Eligible studies

**Figure 1:**
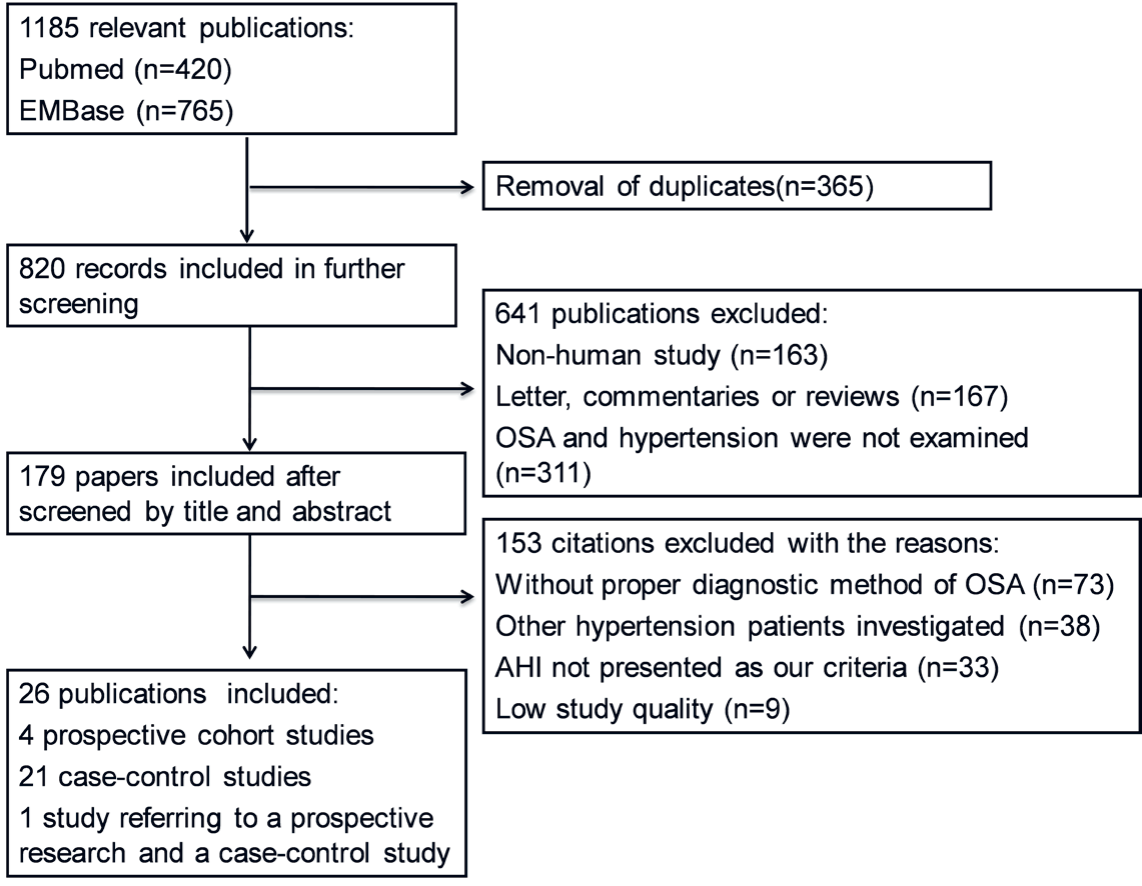
Flowchart of the study selection.

Our initial screen returned 15 031 publications in English, of which 4314 were from Pubmed and 10 717 were from Embase. YZ and WY independently excluded 13 846 ineligible articles through reviewing the titles, leaving 1185 articles for further abstract review. Among them, 26 publications with 51 623 participants (28 314 men, 23 309 women; mean age 51.8 years) met all the inclusion criteria and were enrolled in meta-analysis [1,2,8,12-34]. Among those 1 159 publications excluded at the last round: 365 were duplicate articles, 311 studies did not refer to OSA or hypertension according to the current definitions, 167 were not original studies, 42 studies were not designed as required method, 73 studies did not have appropriate diagnostic criteria for OSA patients, 38 studies investigated other types of hypertension, and 163 were not human-based research. The flowchart for the process of screening articles is shown in **Figure 1**. Among all the enrolled publications, 21 articles reported case-control comparison in cross-sectional studies or baseline data of cohort studies [1,2,12-18,20,22-32], Four were prospective studies [19,21,33,34], and one article reported both a baseline data comparison and a prospective study [8]. In terms of the study topics, six studies investigated the relationship between OSA and resistant hypertension [1,12-16], and 20 studies investigated the association of OSA with essential hypertension [2,8,17-34]. Individual characteristics of overall studies are summarized in **Table 1** and Table S4 in **Online Supplementary Document(Online Supplementary Document)**.

**Table 1:** Characteristics of included studies

Author	Year	Type of HTN	Country	Case	Mean age	Matched Control	Gender	Duration of Follow-up
Walia [1]	2014	R-HTN	United States	Hospital-based OSA	Case:63.4 ± 6.9	NO	Case:81/28	NA*
					Con:62.5 ± 7.4		Con: 126/49	
Wu [12]	2016	R-HTN	China	Hospital-based OSA	54.3 ± 3.0	NO	Case:365/87	NA†
							Con: 166/50	
Abdel-Kader [13]	2012	R-HTN	United States	Community-based R-HTN	60.0 ± 7.2	NO	113/111	NA*
Drager [14]	2009	R-HTN	Brazil	Hospital-based OSA	Case:51 ± 10	NO	Case:30/25	NA†
					Con:40 ± 10		Con: 22/22	
Gonçalves [15]	2007	R-HTN	Brazil	Hospital-based R-HTN	Case:59 ± 7	NO	Case:21/42	NA†
					Con: 59 ± 7		Con: 23/40	
Ruttanaumpawan [16]	2009	R-HTN	Canada	Hospital-based R-HTN	Case:60.1 ± 1.8	YES	Case:12/10	NA†
					Con: 56.5 ± 1.6		Con: 26/16	
Nieto [2]	2003	E-HTN	United States	Community-based OSA	>40	NO	2894/3238	NA*
Bartel [17]	1995	E-HTN	South Africa	Hospital-based E-HTN	Case:50.6 ± 8.0	YES	Case:4/16	NA†
					Con:49.6 ± 8.7		Con: 4/16	
Crocker [18]	1989	E-HTN	Australia	Hospital-based OSA	51.5 ± 14	NO	Case:96/4	NA†
							Con: 78/22	
Peppard [19]	2000	E-HTN	Australia	Hospital-based OSA	51 ± 8	NO	504/389	4 years
Durán [20]	2001	E-HTN	Spain	Community-based OSA	Case: M:52.0 ± 9.9, F:55.5 ± 11.3	NO	Case:255/135	NA†
					Con: M:47.4 ± 10.8, F:46.9 ± 0.5		Con: 69/96	
Guillot [21]	2013	E-HTN	France	Community-based OSA	Case:68.0 ± 1.1	NO	Case:34/39	3 years
					Con: 68.1 ± 0.9		Con:105/194	
Haas [22]	2005	E-HTN	United States	Community-based OSA age 40 to 59	52.2 ± 5.3	NO	1132/1345	NA*
Haas [22]	2005	E-HTN	United States	Community-based OSA>60 years	70.2 ± 6.9	NO	1752/1891	NA*
Appleton [8] (study-a)	2016	E-HTN	Australia	Community-based OSA	58.0 ± 10.7	NO	448/0	NA†
Appleton [8] (study-b)	2016	E-HTN	Australia	Community-based OSA		NO	110/0	56 months
Kapur [23]	2010	E-HTN	United States	Community-based E-HTN	62.6 ± 10.3	NO	2853/3192	NA*
Li [24]	2015	E-HTN	China	Hospital-based OSA with chronic insomnia	43.0 ± 12.1	NO	409/451	NA†
Mokhlesi [25]	2014	E-HTN	United States	Community-based HTN	53 ± 10	NO	4203/182	NA*
Priou [26]	2014	E-HTN	France	Community-based OSA	54.3 ± 13.5	NO	955/544	NA†
Redline [27]	2014	E-HTN	United States	Community-based OSA	M:40.3 ± 0.3, F:41.8 ± 0.3	NO	5747/8693	NA*
Smith [28]	2014	E-HTN	Canada	Community-based OSA	M:46.7 ± 0.4, F:50.5 ± 0.9	YES	Case:599/174	NA†
							Con:599/174	
Xie [29]	2011	E-HTN	United States	OSA from drivers	Case:46.73 ± 9.82	NO	Case:108/0	NA†
					Con:43.63 ± 11.68		Con: 1655/0	
Young [30]	1997	E-HTN	United States	Community-based OSA	30-60	NO	617/443	NA*
Yusoff [31]	2010	E-HTN	Malaysia	OSA from drivers	Case:45.4 ± 7.0	NO	Case:128/0	NA†
					Con:42.5 ± 7.6		Con: 161/0	
Zou [32]	2012	E-HTN	United States	E-Community-based HTN	Case:63 ± 6	NO	Case:76/85	NA*
					Con: 60 ± 7		Con: 97/86	
O’Connor [33]	2009	E-HTN	United States	Community-based OSA	59.6 ± 10.3	NO	1103/1367	5 years
Chan [34]	2016	E-HTN	Singapore	Hospital-based OSA	Case:57.1 ± 9.7	NO	534/53	2.5 years
					Con:57.0 ± 9.6			

R-HTN – Resistant hypertension, E-HTN – Essential hypertension, OSA – obstructive sleep apnea, Con – control group

*Baseline data in cohorts.

†Cross-sectional designed case-control study.

### Association between OSA and resistant hypertension

**Figure 2:**
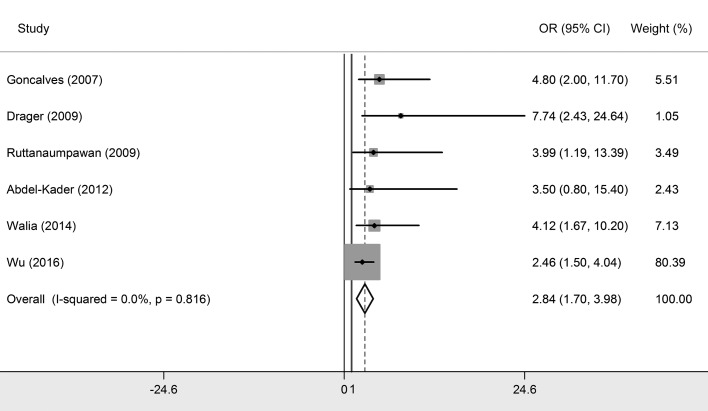
Forest plot of the association between resistant hypertension and obstructive sleep apnea (OSA).

We included six studies that explored the relationship between OSA and resistant hypertension [1,12-16], with five studies conducted among Caucasians and one study was among Asians. The synthesized results showed that OSA was significantly associated with resistant hypertension with a pooled OR of 2.842 (95% CI = 1.703-3.980, *P* < 0.05) (**Figure 2** and **Table 2**), which indicated that OSA participants had an extra 1.842-fold risk for resistant hypertension prevalence compared to non-OSA participants. Subgroup analysis exposited that this association was more outstanding among Caucasian populations (OR = 4.406, 95% CI = 1.835-6.977).

**Table 2:** Meta-analysis of association between OSA and hypertension

Type of HTN	Category of OSA	Subgroup	Effect size				Heterogeneity test		
**Pooled OR**	**95% CI**	***P***		**I^2^ (%)**	**Q**	***P***
**Resistant HTN**	OSA/non-OSA	Asian	2.460	1.500-4.040	<0.05		-	-	-
	OSA/non-OSA	Caucasian	4.406	1.835-6.977	<0.05		0	0.47	0.977
	OSA/non-OSA	Overall	2.842	1.703-3.980	<0.05		0	2.23	0.816
**Essential HTN**	Mild OSA	Prospective	1.038	0.808-1.267	>0.05		64.8	5.69	0.058
	Mild OSA	Non-prospective	1.210	1.112-1.309	<0.05		0	3.60	0.825
	Mild OSA	Overall	1.184	1.093-1.274	<0.05		10.1	11.13	0.348
	Moderate OSA	Caucasian	1.315	1.197-1.433	<0.05		8.0	16.30	0.362
	Moderate OSA	Prospective	1.224	0.917-1.530	>0.05		12.6	3.43	0.329
	Moderate OSA	Non-prospective	1.332	1.204-1.456	<0.05		5.4	12.69	0.392
	Moderate OSA	Overall	1.316	1.198-1.434	<0.05		3.2	16.53	0.416
	Severe OSA	Caucasian	1.549	1.275-1.824	<0.05		0	7.49	0.586
	Severe OSA	Prospective	1.505	0.998-2.013	>0.05		0	1.00	0.608
	Severe OSA	Non-prospective	1.584	1.258-1.909	<0.05		10.2	7.80	0.351
	Severe OSA	Overall	1.561	1.287-1.835	<0.05		0	8.86	0.546
**Essential HTN**	OSA/non-OSA*	Asian	1.583	1.160-2.007	<0.05		0	0.32	0.850
	OSA/non-OSA*	Caucasian	1.928	1.600-2.256	<0.05		47.0	7.55	0.110
	OSA/non-OSA*	Prospective	1.475	1.035-1.914	<0.05		0	0.02	0.881
	OSA/non-OSA*	Non-prospective	1.972	1.651-2.294	<0.05		19.8	6.23	0.284
	OSA/non-OSA*	Overall	1.799	1.539-2.058	<0.05		26.0	9.46	0.221

HTN – hypertension, OSA – obstructive sleep apnea, OR – odds ratio; CI – confidence interval

*Meta-analysis of six studies only divided subjects into OSA and non-OSA groups.

The heterogeneity test showed no significant heterogeneity generated across all primary studies (I^2^ = 0%, Q = 2.23, *P* = 0.816).

### Association between OSA and essential hypertension

**Figure 3:**
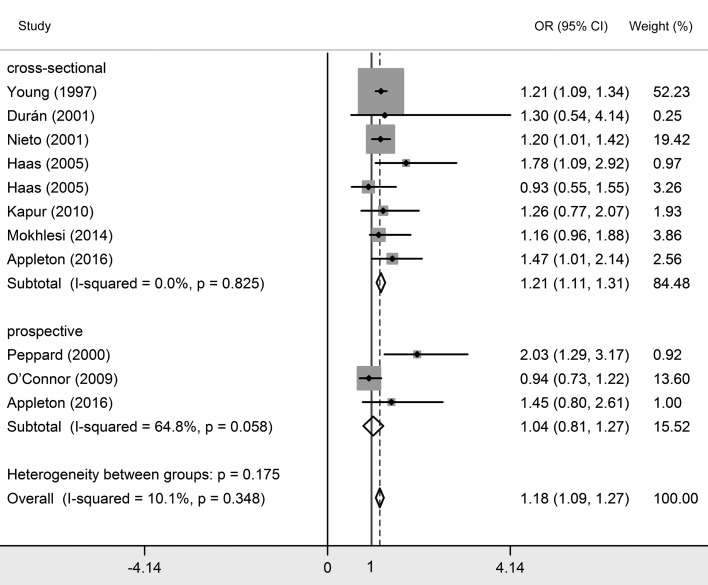
Forest plots of the association between essential hypertension and mild obstructive sleep apnea (OSA).

**Figure 4:**
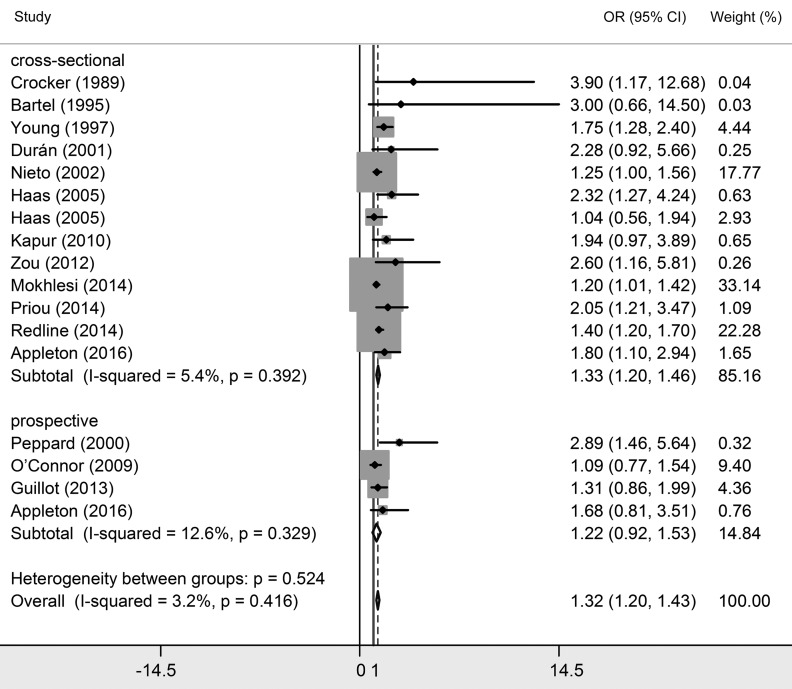
Forest plots of the association between essential hypertension and moderate obstructive sleep apnea (OSA).

**Figure 5:**
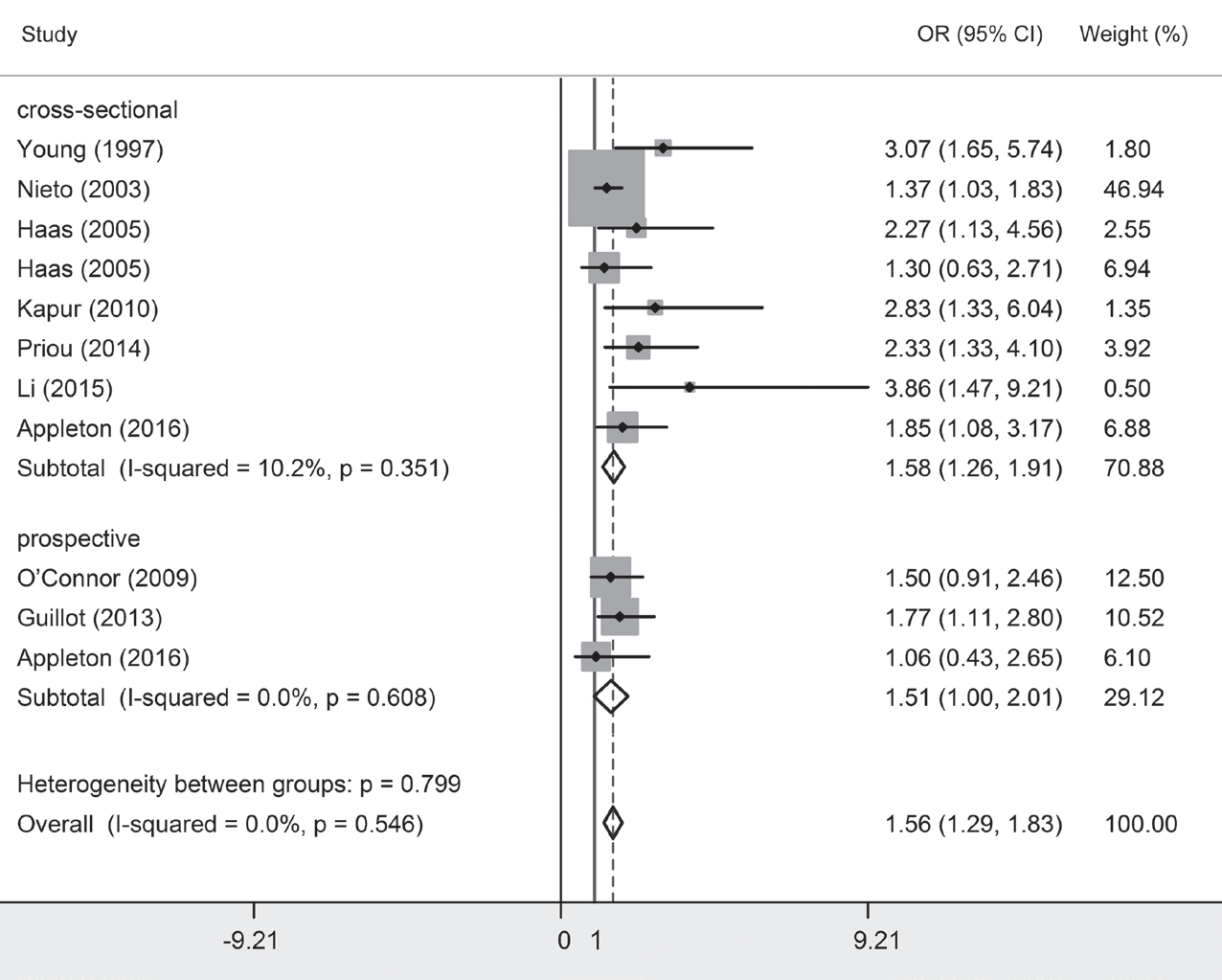
Forest plots of the association between essential hypertension and severe obstructive sleep apnea OSA).

Twenty studies investigated the relationship between OSA and essential hypertension [2,8,17-34]. Among them, 16 studies classified OSA patients into three grades based on AHI their scores, which were mild OSA (AHI>5), moderate OSA (AHI>15) and severe OSA (AHI>30). Since natural differences exists between mild OSA, moderate OSA and severe OSA, results were interpreted separately. As shown in **Table 2** and **Figures 3 to 5**, essential hypertension was significantly associated with OSA, in terms of mild OSA (OR = 1.184, 95% CI = 1.093-1.274, *P* < 0.05), moderate OSA (OR = 1.316, 95% CI = 1.198-1.434, *P* < 0.05), and severe OSA (OR = 1.561, 95% CI = 1.287-1.835, *P* < 0.05). These results showed the trend of that the more serious OSA is, the higher hypertension risk occurs. Subgroup analyses were conducted based on study design (prospective/non-prospective) and ethnicity. As shown in **Table 2**, no significant results were found in studies with prospective study design, which outlined the pooled ORs (95% CIs) of 1.038 (0.808-1.267) for mild OSA, 1.224 (0.917-1.530) for moderate OSA, and 1.505 (0.998 – 2.013) for severe OSA. The heterogeneity test illustrated that I^2^ values were 10.1% (*P* = 0.348) in mild OSA, 3.2% (*P* = 0.416) in moderate OSA, and 0% (*P* = 0.546) in severe OSA meta-analyses, indicating no significant heterogeneity in each group.

For the seven studies dividing participants only into OSA and non-OSA groups [8,24,26,28,29,31,34], the pooled OR was 1.799 (95% CI = 1.539-2.058, *P* < 0.05), demonstrating that OSA was associated with essential hypertension (Figure S1 and Table S2 in **Online Supplementary Document(Online Supplementary Document)**). The subgroup analysis based on ethnicity showed significant results among Asians (OR = 1.583, 95% CI = 1.160-2.007) and Caucasians (OR = 1.928, 95% CI = 1.600-2.256). Additionally, no significant heterogeneity was found (I^2^ = 26.0%, *P* = 0.221). Four studies in the three articles reported the relationship between OSA and essential hypertension among male participants [2,8,33]. The combined result of these four studies suggested that the association between OSA and essential hypertension was relatively stronger among males (OR = 1.698, 95% CI = 1.319-2.077).

### Publication bias

The funnel plot analysis, followed by Egger’s test, was applied to detect the potential publication bias. Significant publication bias existed in the meta-analyses of moderate OSA and severe OSA participants, and the meta-analysis of resistant hypertension (**Table 3** and Figures S7-S11 in **Online Supplementary Document(Online Supplementary Document)**). The Trim and Fill analysis was performed to eliminate the effect of publication bias on meta-analysis. As shown in **Table 3** and Figures S12-S14 in **Online Supplementary Document(Online Supplementary Document)**, the pooled ORs (95% CIs) were 1.327 (1.222-1.441) for moderate OSA and 1.593 (1.369-1.853) for severe OSA meta-analyses, and 1.575 (1.117-2.221) for resistant hypertension.

**Table 3:** Analysis on publication bias and results of Trim and Fill method analysis

Type of HTN	Category of OSA	Egger’s test		Trim and fill method analysis		
***t***	***P***	**Pooled OR**	**95% CI**	***P***
Resistant HTN	OSA/non-OSA	2.36	0.078	1.575	1.117-2.221	0.010
Essential HTN	Mild OSA	0.92	0.380	1.187	1.103-1.276	<0.001
Essential HTN	Moderate OSA	3.91	0.001	1.327	1.222-1.441	<0.001
Essential HTN	Severe OSA	1.98	0.079	1.593	1.369-1.853	<0.001
Essential HTN	OSA/non-OSA	0.87	0.420	1.356	1.184-1.554	<0.001

HTN – hypertension, OSA – obstructive sleep apnea, OR – odds ratio, CI – confidence interval

### Sensitivity analysis

To evaluate the robustness of the results, sensitivity analyses were undertaken by sequentially removing each study. Consequently, no obvious change was generated on omission of any individual study (Figure S2-S6 in **Online Supplementary Document(Online Supplementary Document)**), confirming that the results of our meta-analyses were stable. This coincided with the results of heterogeneity tests.

## DISCUSSION

This systematic review and meta-analysis was conducted in an effort to clarify the association between OSA and hypertension, including both essential and resistant hypertension. Our findings suggest that OSA confers a significant association with both essential and resistant hypertension. Moreover, OSA is associated with mild OSA, moderate OSA and severe OSA with a stepwise increased degree.

OSA are acknowledged to be associated with hypertension [6,7]. Nevertheless, the potential mechanisms underlying the associations have not been well elucidated. Several potential explanations may help us to understand the association of OSA and hypertension. OSA induces intermittent hypoxemia, similarly to hypoxia/reperfusion injury, and causes oxidative stress, leading to dysfunction of vascular endothelium [35]. Meanwhile, excessive outflow of sympathetic vasoconstrictor together with diminished nitric oxide bioavailability plays a role in blood pressure elevation [36,37]. In addition, episodes of OSA up-regulate sympathetic excitation which acts on the chemoreflex and may consequently result in hypertension [10,38]. In clinical observations, sympathetic nervous activity, reflected by 24-hour urinary catecholamine excretion, is increased in individuals with sleep-disordered breathing [39]. The data from relevant surveys revealed that the prevalence rate of OSA is 70-83% in resistant hypertensive patients [40]. It is also suggested that untreated OSA may lead to reduced effectiveness of medications through pharmacokinetic or chronotherapeutic effects, activating a pathway of resistance to antihypertensive drugs [1]. In the present study, we found that OSA strongly predisposes to resistant hypertension by combining six relevant studies. The pooled OR of the causal association in Caucasians is 4.406, which indicates Caucasians with OSA suffer more from uncontrolled hypertension.

OSA is also considered to be associated with essential hypertension and the consequences of cardiovascular diseases. However, the results are not entirely consistent, meanwhile more than eight studies have generated non-significant results [2,8,13,17,20,22-23,33]. This meta-analysis, involving 20 original studies in 19 articles, demonstrates that OSA increases the risk of essential hypertension in a dose-response manner. Although the dose-response meta-analysis has not been conducted owing to the lack of current available data, the trend of pooled ORs shows obviously that the more serious OSA is, the higher hypertension risk occurs.

Gender is indispensable factor in the analysis of demographic data. Nonetheless, seldom studies reported the association between OSA and hypertension in specific gender. O’Connor et al. reported that female patients with moderate and severe OSA have higher prevalence of essential hypertension than male [33], while, Nieto *et al* reported a lower OR in severe OSA women before [2]. After combining four studies reported in three articles [8,29,31], our finding enlightens that men suffer from higher incidence of essential hypertension than women, as well the association in Caucasians is greater than that in Asians. These differences may be explained by the larger proportion of overweight and obesity among Caucasians and also among men [18]. This finding is consistent with a recent study by Cano-Pumarega et al., despite the fact that their study did not diagnose OSA with polysomnography and AHI [41]. Regarding ethnic sub-group analysis, the different results may subject to genetic backgrounds, environmental factors and genetic-environmental interaction [42,43].

Heterogeneity may result from diversity of clinical study design or statistical methodology, and the detection of heterogeneity is essential for meta-analysis. No significant heterogeneity was found in the present study, indicating high compatibility of our results. Care must be taken in the adjustment of potential confounding factors in the statistical analysis of clinical studies. Obviously, obesity and age are both contributing factors to OSA and hypertension. Although investigators suggested that the impact of body mass index (BMI) on OSA is quite modest and adjustment for BMI might be an over-adjustment [2,32], we have synthesized the OR adjusted for both age and BMI in order to estimate the risk more precisely.

Publication bias, a critical issue in systematic review, mainly results from the tendency that studies with positive results are more likely to be published than those with negative findings. Significant publication bias exists in our meta-analyses of moderate OSA, severe OSA and essential hypertension. To reduce potential confounding of publication bias, we carried out the Trim and Fill analysis. According to the results of Trim and Fill analysis, the pooled ORs remained statistically significant for the association between moderate OSA and essential hypertension, the association between severe OSA and essential hypertension, as well as the association between OSA and resistant hypertension.

Comparing to low specificity of sleep questionnaires, AHI is an accurate parameter for determining OSA [14], which is defined as the number of episodes of apneas or hypopneas in 1 hour of sleep, recorded with polysomnography test [2]. One of inclusion criteria in this study is the usage of AHI as OSA diagnostic index, this obviously limits the number of studies to be included.

This study has certain limitations that are common in systematic reviews meta-analyses of descriptive studies: First, most of the original studies were based on cross-sectional designed investigation, which may limit the argumentation intensity of our conclusion. Second, the subgroup analyses based on age were not performed because of no primary data available from the included studies. Only 5 prospective studies [8,19,21,33,34] meeting the inclusion criteria were included, and the pooled results of the studies showed no statistical significance of association between OSA and hypertension. However, the number of included studies/patients is small. More prospective studies need to be conducted to clarify the relationship between OSA and hypertension.

The present study demonstrates that OSA is related to an increased risk of resistant hypertension, with a stronger association among Caucasian populations and male OSA patients. Mild, moderate and severe OSA are associated essential hypertension, as well a dose-response manner is manifested.

Our interpretation of synthesized epidemiological evidence strengthens the acceptance of OSA as a risk factor of hypertension, in term of essential hypertension and resistant hypertension. Given the underlying mechanisms have been explained, the key remaining questions are how does OSA mediate hypertension and how can we implement specific interventions to reduce hypertension induced by OSA. More studies should be conducted to examine the mechanisms of hypertension regulation by OSA, especially among medication-resistant hypertension patients.
