# Supplementary material for: Association of obstructive sleep apnea with hypertension: A systematic review and meta-analysis
Source: J Glob Health. 2018 Feb 10;8(1):010405. doi: 10.7189/jogh.08.010405 (PMC5825975; doi:10.7189/jogh.08.010405)
Supplement: Online Supplementary Document [file jogh-08-010405-s001.pdf]

Online Supplementary Document

Hou et al. Association of obstructive sleep apnea with hypertension: A systematic review and meta-analysis

J Glob Health 2018;8:010405

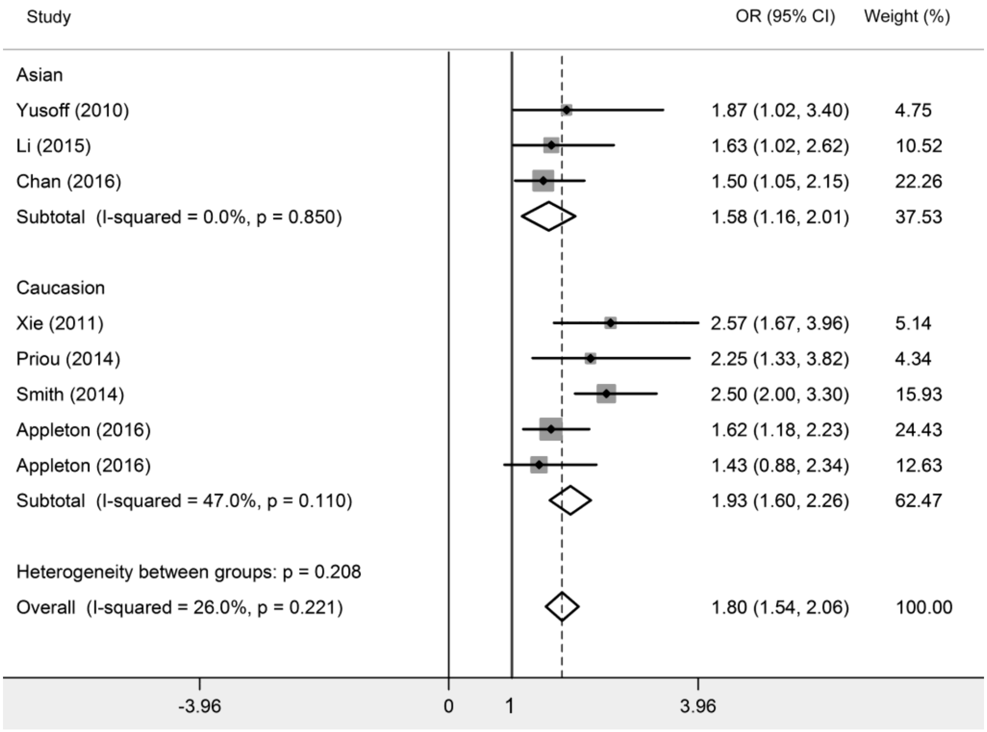

Figure S1. Forest plot of the association between essential hypertension and OSA/non-OSA

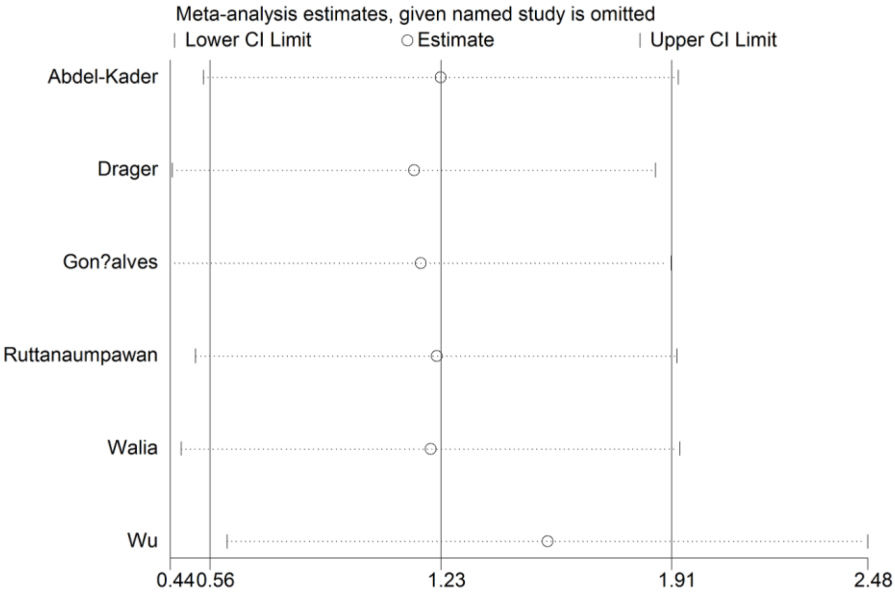

Figure S2. Sensitivity analysis of association between resistant hypertension and OSA

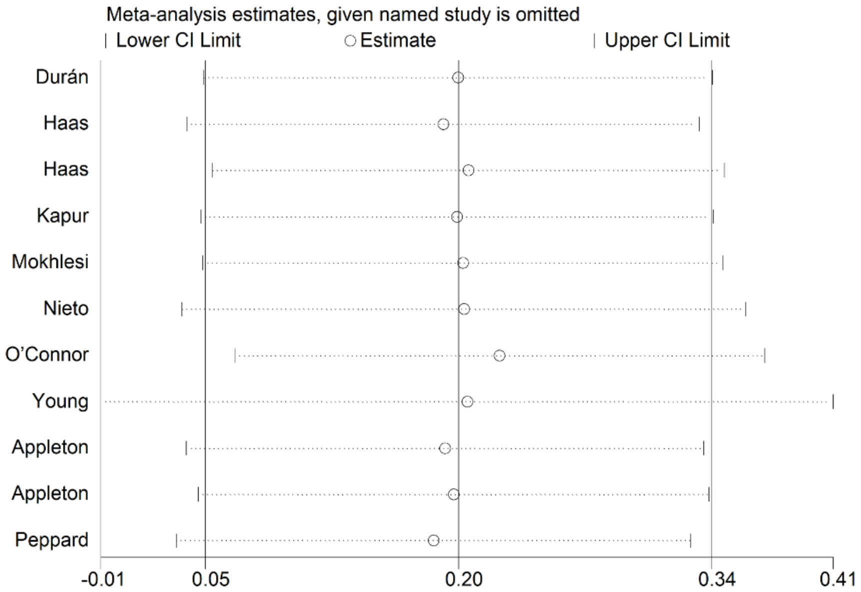

Figure S3. Sensitivity analysis of the association between essential hypertension and mild OSA

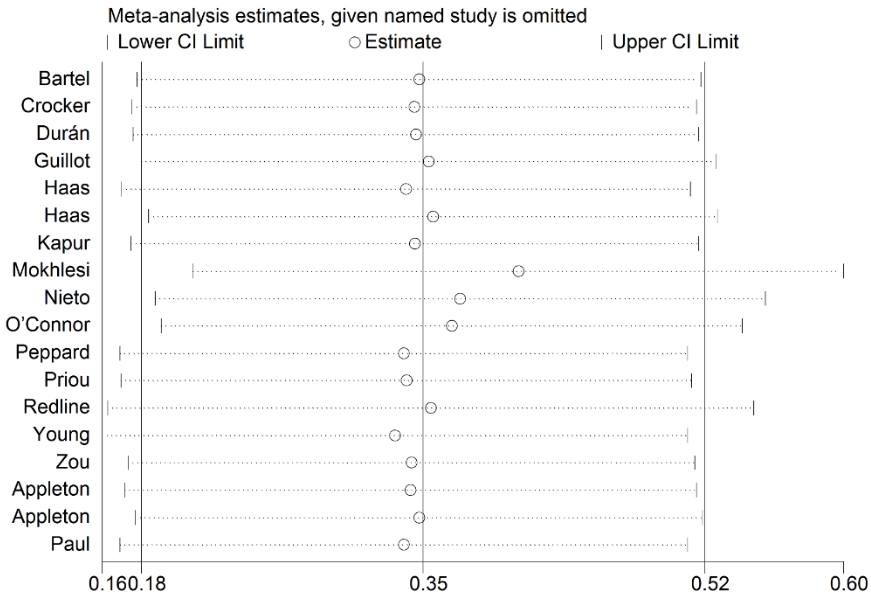

Figure S4. Sensitivity analysis of the association between essential hypertension and moderate OSA

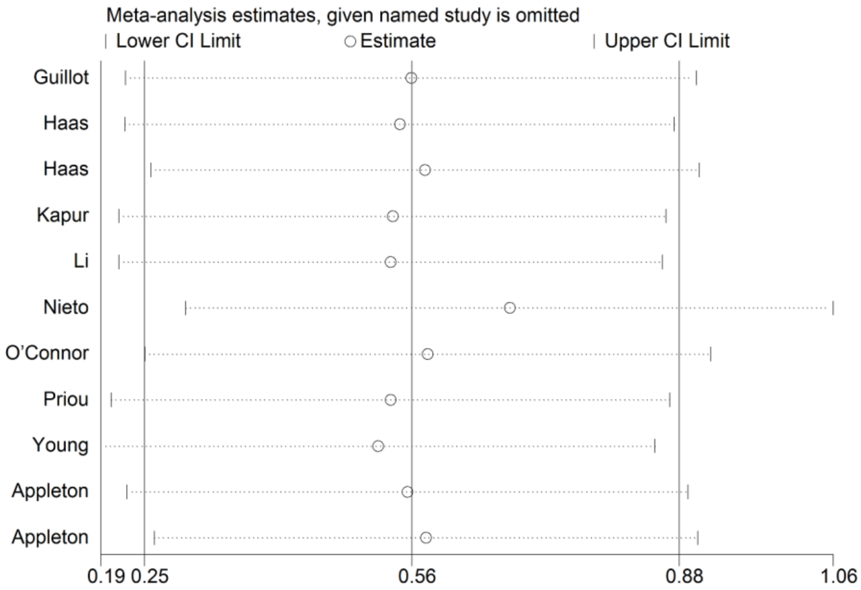

Figure S5. Sensitivity analysis of the association between essential hypertension and severe OSA

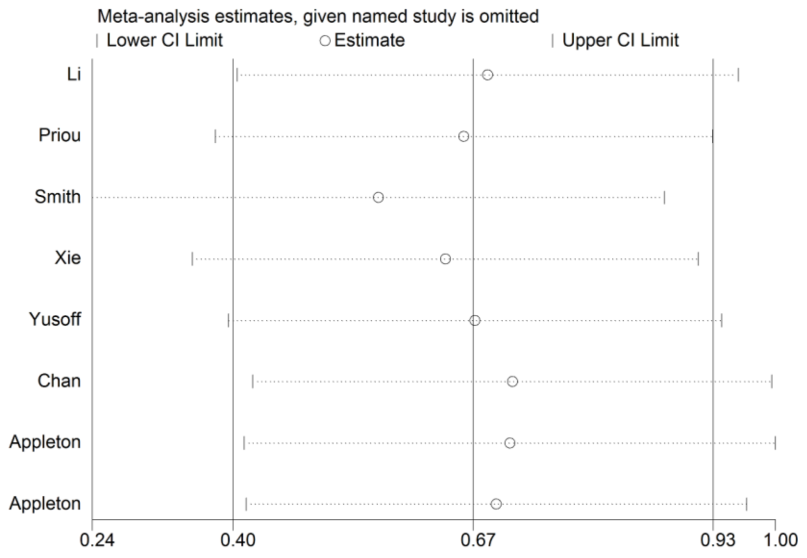

Figure S6. Sensitivity analysis of the association between essential hypertension and OSA/non-OSA

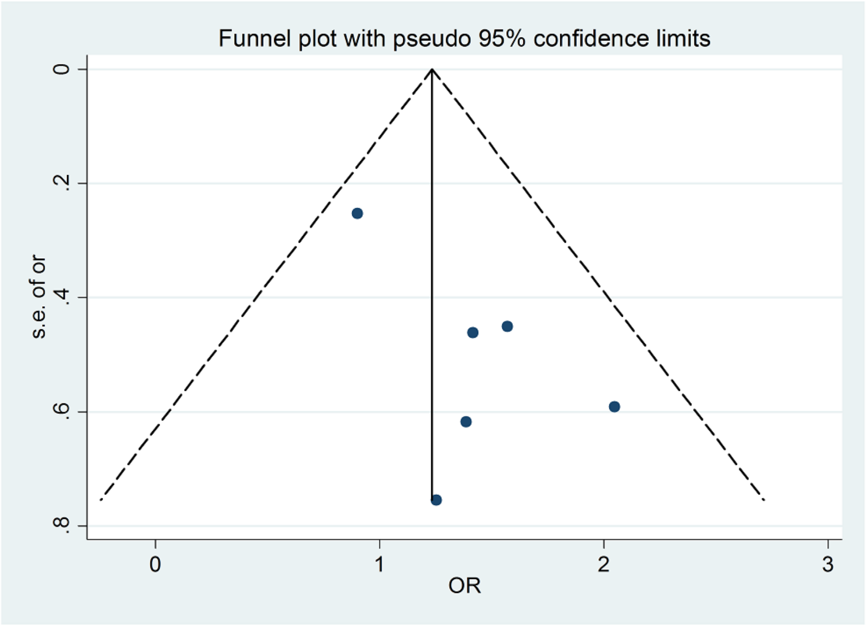

Figure S7. Funnel plot of association between resistant hypertension and OSA

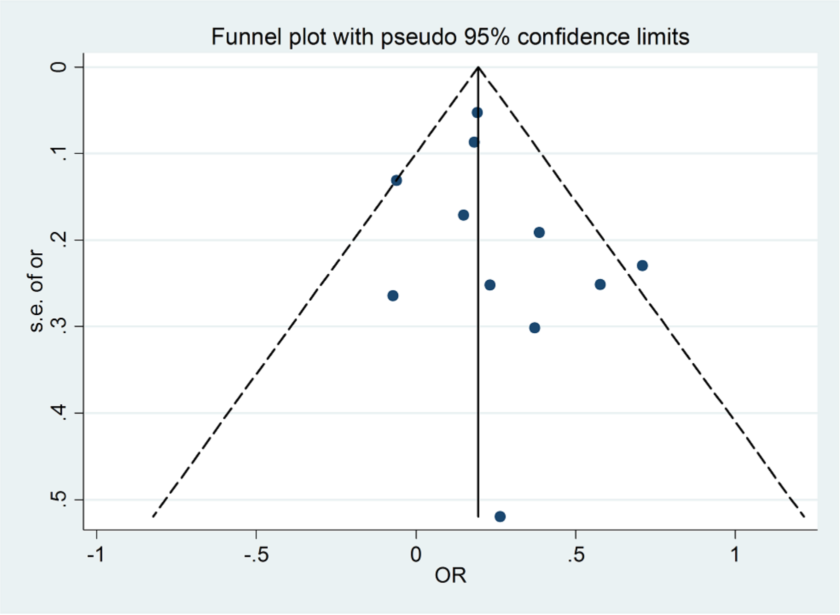

Figure S8. Funnel plot of the association between essential hypertension and mild OSA

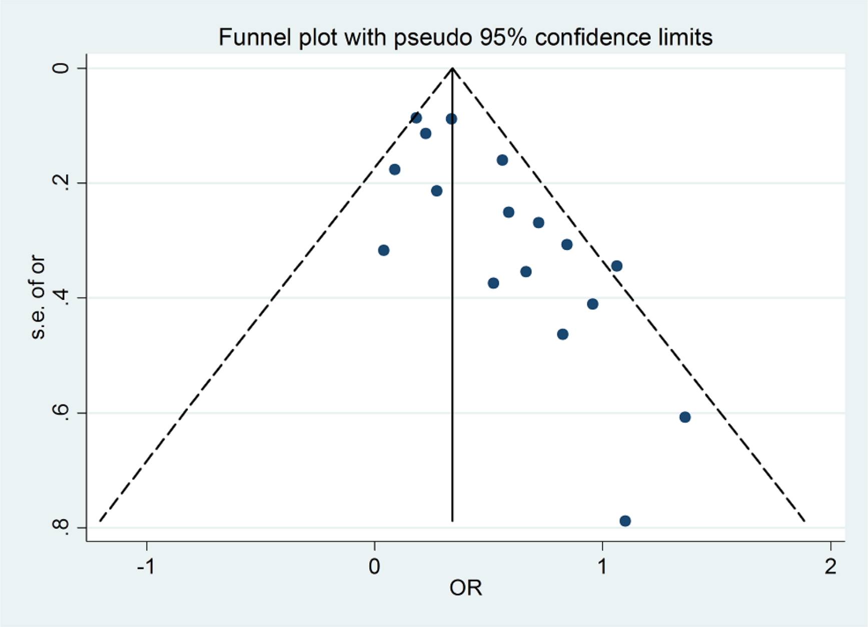

Figure S9. Funnel plot of the association between essential hypertension and moderate OSA

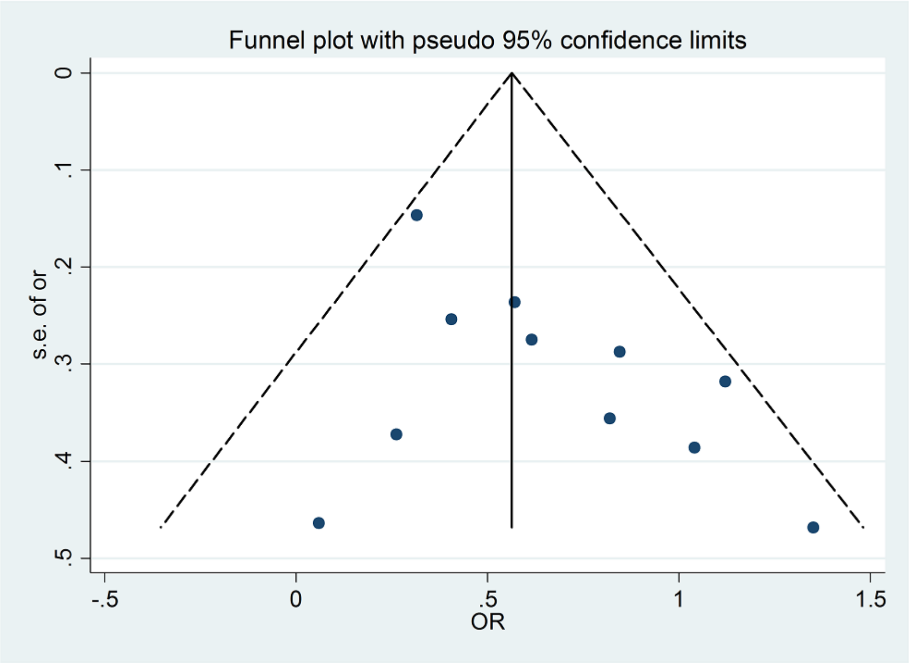

Figure S10. Funnel plot of the association between essential hypertension and severe OSA

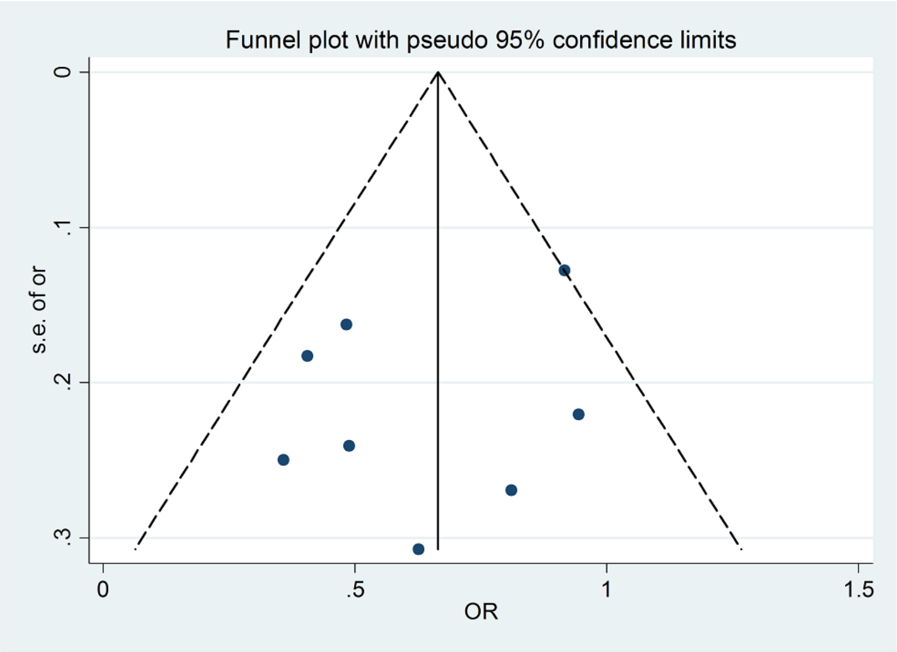

Figure S11. Funnel plot of the association between essential hypertension and OSA/non-OSA

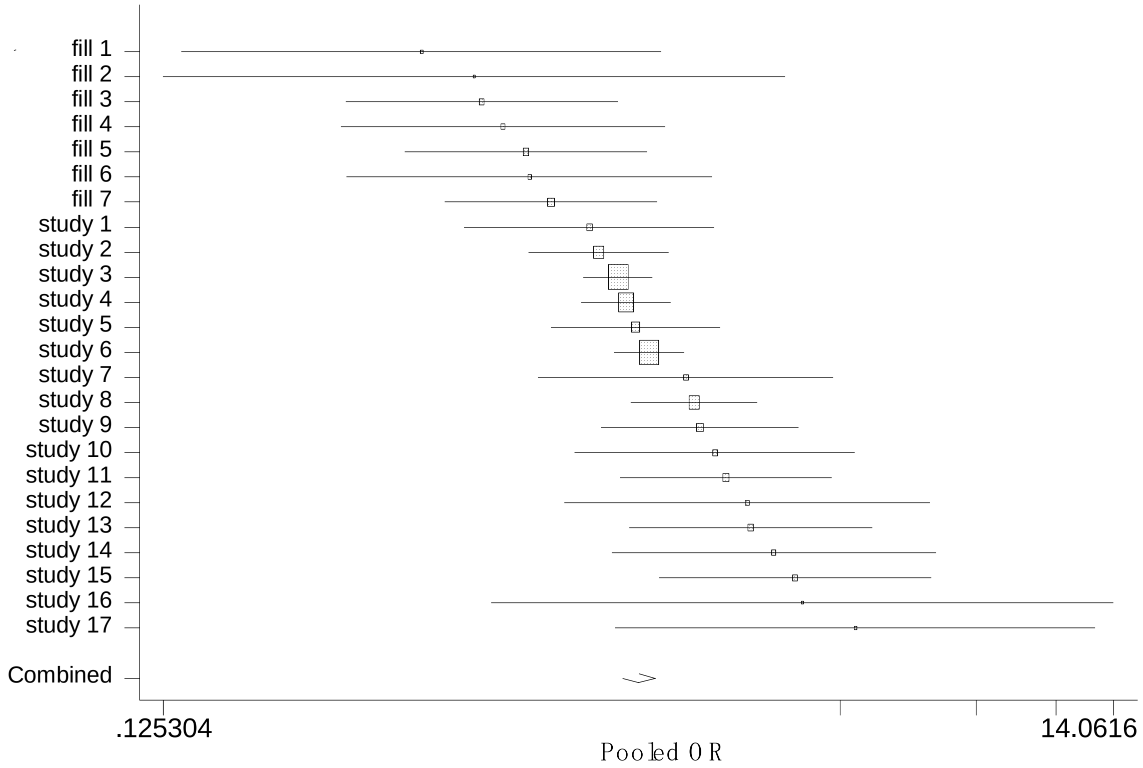

Figure S12. Trim and Fill method analysis of the association between essential hypertension and moderate OSA

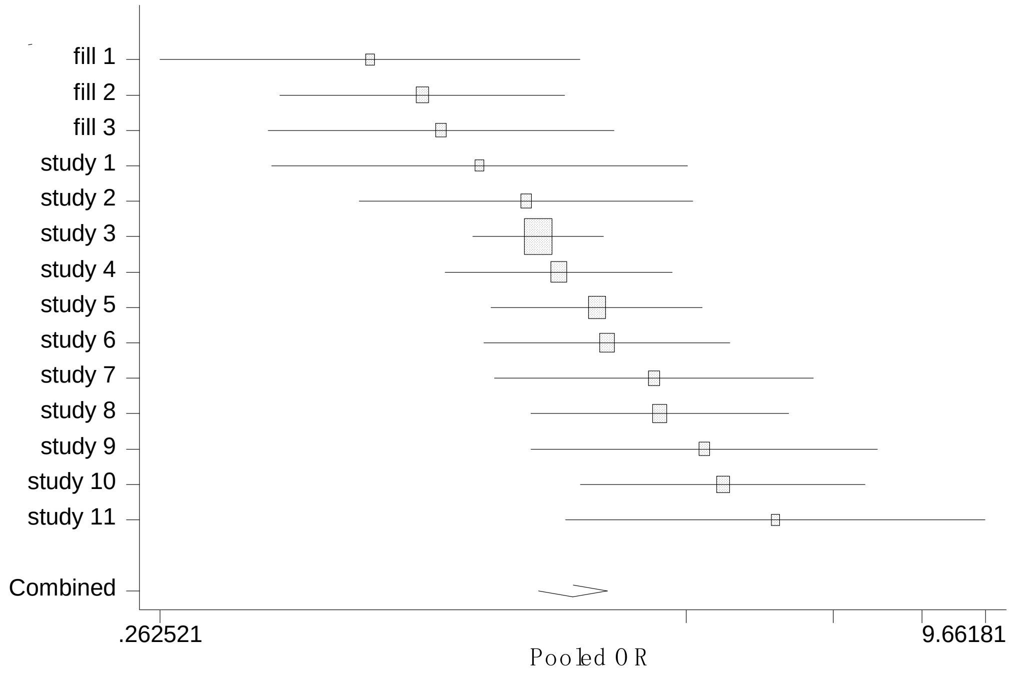

Figure S13. Trim and Fill method analysis of the association between essential hypertension and severe OSA

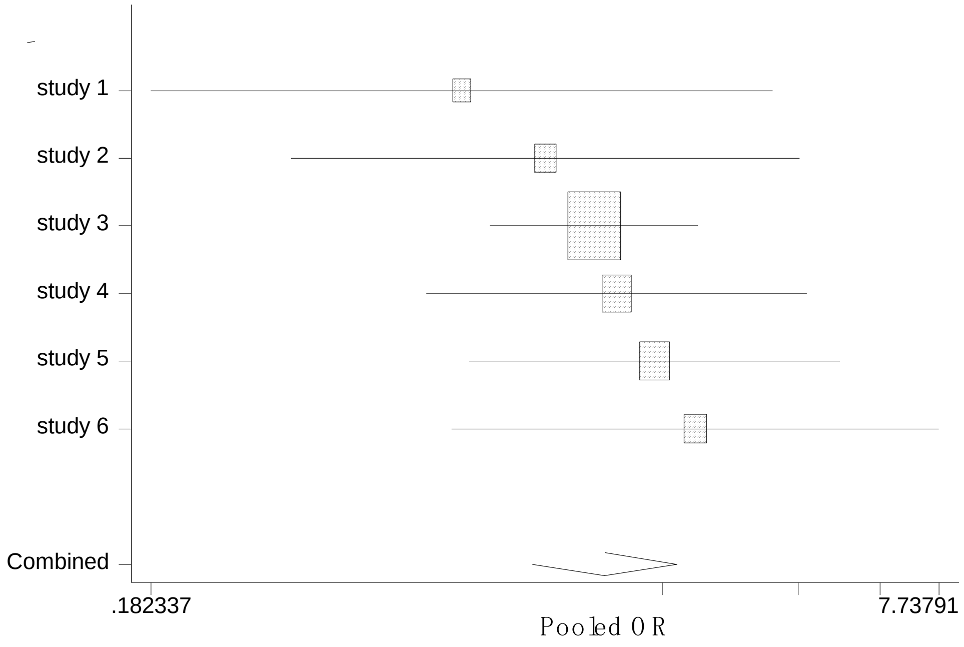

Figure S14. Trim and Fill method analysis of the association between resistant hypertension and OSA

Table S1. The checklist of PRISMA Statement

Section/Topic	Checklist Item	Reported or not
TITLE		
Title	Identify the report as a systematic review, meta-analysis, or both.	Y
ABSTRACT		
Structured summary	Provide a structured summary including, as applicable: background; objectives; data sources; study eligibility criteria, participants, and interventions; study appraisal and synthesis methods; results; limitations; conclusions and implications of key findings; systematic review registration number.	Y
INTRODUCTION		
Rationale	Describe the rationale for the review in the context of what is already known.	Y
Objectives	Provide an explicit statement of questions being addressed with reference to participants, interventions, comparisons, outcomes, and study design (PICOS).	Y
METHODS		
Protocol and registration	Indicate if a review protocol exists, if and where it can be accessed (e.g., Web address), and, if available, provide registration information including registration number.	Y
Eligibility criteria	Specify study characteristics (e.g., PICOS, length of follow-up) and report characteristics (e.g., years considered, language, publication status) used as criteria for eligibility, giving rationale.	Y
Information sources	Describe all information sources (e.g., databases with dates of coverage, contact with study authors to identify additional studies) in the search and date last searched.	Y
Search	Present full electronic search strategy for at least one database, including any limits used, such that it could be repeated.	Y
Study selection	State the process for selecting studies (i.e., screening, eligibility, included in systematic review, and, if applicable, included in the meta-analysis).	Y
Data collection process	Describe method of data extraction from reports (e.g., piloted forms, independently, in duplicate) and any processes	Y

	for obtaining and confirming data from investigators.	
Data items	List and define all variables for which data were sought (e.g., PICOS, funding sources) and any assumptions and simplifications made.	Y
Risk of bias in individual studies	Describe methods used for assessing risk of bias of individual studies (including specification of whether this was done at the study or outcome level), and how this information is to be used in any data synthesis.	Y
Summary measures	State the principal summary measures (e.g., risk ratio, difference in means).	Y
Synthesis of results	Describe the methods of handling data and combining results of studies, if done, including measures of consistency (e.g., I2) for each meta-analysis.	Y
Risk of bias across studies	Specify any assessment of risk of bias that may affect the cumulative evidence (e.g., publication bias, selective reporting within studies).	Y
Additional analyses	Describe methods of additional analyses (e.g., sensitivity or subgroup analyses, meta-regression), if done, indicating which were pre-specified.	Y
RESULTS		
Study selection	Give numbers of studies screened, assessed for eligibility, and included in the review, with reasons for exclusions at each stage, ideally with a flow diagram.	Y
Study characteristics	For each study, present characteristics for which data were extracted (e.g., study size, PICOS, follow-up period) and provide the citations.	Y
Risk of bias within studies	Present data on risk of bias of each study and, if available, any outcome-level assessment (see Item 12).	Y
Results of individual studies	For all outcomes considered (benefits or harms), present, for each study: (a) simple summary data for each intervention group and (b) effect estimates and confidence intervals, ideally with a forest plot.	Y
Synthesis of results	Present results of each meta-analysis done, including confidence intervals and measures of consistency.	Y
Risk of bias across studies	Present results of any assessment of risk of bias across studies (see Item 15).	Y
Additional analysis	Give results of additional analyses, if done (e.g., sensitivity or subgroup analyses, meta-regression [see Item 16]).	Y
DISCUSSION		Y
Summary of evidence	Summarize the main findings including the strength of evidence for each main outcome; consider their relevance to key groups (e.g., health care providers, users, and policy makers).	Y

Limitations	Discuss limitations at study and outcome level (e.g., risk of bias), and at review level (e.g., incomplete retrieval of identified research, reporting bias).	Y
Conclusions	Provide a general interpretation of the results in the context of other evidence, and implications for future research.	Y
FUNDING		
Funding	Describe sources of funding for the systematic review and other support (e.g., supply of data)	Y

Y: the item was reported in article, N: the item was not reported.

Table S2. Scale for quality assessment based on PRISMA statement and MOOSE guideline

Criteria	Score
**Representativeness of cases**	
Characteristics of participants were described.	1
Consecutive/randomly selected from case population was clearly defined.	1
Eligible patients are similar to controls, in term of age, gender and other important characteristics.	1
The percentage of loss to follow-up was provided, or the reasons of loss to follow-up were mentioned.	1
**Accuracy of information**	
Methods of variable measurement were offered	1
Definitions of outcome were offered.	1
**Statistical analyses**	
Methods of statistical analyses were adequate to resolve research hypothesis.	1
Multivariate analyses were performed.	1
**Final question**	

If there were any other important flaws in the design, the study would be not included.

Table S3. Quality score of each study

Author	Item 1	Item 2	Item 3	Item 4	Item 5	Item 6	Item 7	Item 8	Sum
Abdel-Kader	1	0	1	0	1	1	1	1	6
Appleton	1	1	1	1	1	1	1	1	8
Bartel	1	0	1	0	1	1	1	1	6
Chan	1	1	1	0	1	1	1	1	7
Crocker	1	1	1	0	1	1	1	1	7
Drager	1	1	1	0	1	1	1	1	7
Durán	1	1	1	0	1	1	1	1	7
Gonçalves	1	1	1	0	1	1	1	1	7
Guillot	1	1	1	0	1	1	1	1	7
Hass	1	1	1	0	1	1	1	1	7
Wu H	1	0	1	0	1	1	1	1	6
Kapur	1	1	1	0	1	1	1	1	7
Li	1	0	1	0	1	1	1	1	6
Mokhlesi	1	0	1	0	1	1	1	1	6
Nieto	1	1	1	0	1	1	1	1	7
O’Connor	1	1	1	1	1	1	1	1	8
Paul	1	1	1	1	1	1	1	1	8

Peppard	1	1	1	1	1	1	1	1	8
Priou	1	1	1	1	1	1	1	1	8
Redline	1	1	1	1	1	1	1	1	8
Ruttanaumpawan	1	1	1	0	1	1	1	1	7
Smith	1	0	1	0	1	1	1	1	6
Walia	1	1	1	1	1	1	1	1	8
Wu	1	0	1	0	1	1	1	1	6
Xie	1	0	1	1	1	1	1	1	7
Young	1	0	1	1	1	1	1	1	7
Yusoff	1	1	1	0	1	1	1	1	7
Zou	1	1	1	1	1	1	1	1	8

The quality scores were assessed according to items in Table S2.

Table S4. Original data extracted from included studies

Author	Year	n	OR(95%CI)	Adjusted variables
Walia [1]	2014	284	4.12(1.67-10.20)*	Age, gender, race, BMI, smoking, diabetes, CVD.
Wu [12]	2016	668	2.46(1.50-4.04)*	Drinking, age, BMI, smoking, sex, nationality
Abdel-Kader [13]	2012	224	3.5(0.8-15.4)*	Age, gender, race, BMI
Drager [14]	2009	99	7.74(2.43-24.64)*	Age, gender, neck circumference, waist circumference, metabolic syndrome
Gonçalves [15]	2007	126	4.8(2-11.7)*	Age, gender, BMI, HTN duration
Ruttanaumpawan [16]	2009	64	3.99(1.19-13.39)*	BMI, age, gender

Nieto [2]	2003	6132	Mild OSA: 1.20(1.01-1.42)<br>Moderate OSA: 1.25(1-1.56)<br>Severe OSA: 1.37(1.03-1.83)	Age, gender, race, BMI, neck, waist-hip ratio, alcohol use, smoking
Bartel [17]	1995	40	Moderate OSA: 3.00(0.66-14.5)	Age, gender, BMI and neck circumference
Crocker [18]	1989	200	Moderate OSA: 3.9(1.17-12.68)	Age, gender, BMI, accident
Peppard [19]	2000	893	Mild OSA:2.03 (1.29-3.17)<br>Severe OSA:2.89 (1.46-5.64)	BMI, neck and waist circumference, age, sex, use of alcohol and smoking
Durán [20]	2001	555	Mild OSA: 1.3(0.54-4.14)<br>Moderate OSA: 2.28(0.92-5.66)	BMI, neck circumference, alcohol use, and smoking habit
Guillot [21]	2013	372	Moderate OSA: 1.31 (0.86–1.99)<br>Severe OSA: 1.77 (1.11–2.80)	Gender, BMI, diabetes, dyslipidemia
Haas [22]	2005	2477	Mild OSA: 1.78(1.09-2.92)<br>Moderate OSA: 2.32(1.27-4.24)<br>Severe OSA: 2.27(1.13-4.56)	Age, gender, HTN medication use, race, smoking, alcohol use, diabetes, BMI, and waist-hip ratio
Haas [22]	2005	3643	Mild OSA: 0.93(0.55-1.55)<br>Moderate OSA: 1.04(0.56-1.94)<br>Severe OSA: 1.30(0.63-2.71)	Age, gender, HTN medication, race, smoking, alcohol use, diabetes, BMI, and waist-hip ratio
Appleton [8]	2016	448	Mild OSA: 1.47(1.01-2.14)<br>Moderate OSA: 1.80(1.10-2.94)<br>Severe OSA: 1.85(1.08-3.17)	Age, waist circumference, smoking, alcohol; and weight gain over the follow-up period.
Appleton [8]	2016	110	Mild OSA: 1.45 (0.80-2.61)<br>Moderate OSA: 1.68 (0.81-3.51)<br>Severe OSA: 1.06 (0.43-2.65)	Age, waist circumference, smoking, alcohol; and weight gain over the follow-up period
Kapur [23]	2010	6045	Mild OSA: 1.26(0.77-2.07)<br>Moderate OSA: 1.94(0.97-3.89)<br>Severe OSA: 2.83(1.33-6.04)	Age, gender, race, BMI, neck circumference, waist-hip ratio, smoking, alcohol use

Li [24]	2015	860	1.63(1.02-2.62)*	Age, gender, snoring, BMI, heart disease, diabetes, alcohol, smoking, caffeine use
Mokhlesi [25]	2014	4385	Mild OSA: 1.16(0.96-1.41)<br>Moderate OSA: 1.2(1.01-1.42)	Age, gender, race, BMI, waist-hip ratio, smoking status, and alcohol use.
Priou [26]	2014	1499	Non-OSA:1;<br>Moderate OSA: 2.05(1.21–3.47)<br>Severe OSA: 2.33 (1.33–4.10)	Age, gender, obesity, diabetes, depression, smoking, use of thyroid hormones, sleep index, overall arousal index
Redline [27]	2014	14440	Moderate OSA: 1.4(1.2-1.7)	Age, gender, Hispanic/Latino background, education, marital status, cigarette use, alcohol use, site，BMI, waist circumference
Smith [28]	2014	1546	2.5(2-3.3) *	Age, gender, BMI, sleepiness (ESS)
Xie [29]	2011	1763	2.57(1.67-3.96)*	BMI, neck circumference, and minimal oxygen saturation, diabetes, heart disease
Young [30]	1997	1060	Mild OSA: 1.21(1.09-1.34)<br>Moderate OSA: 1.75(1.28-2.4)<br>Severe OSA: 3.07(1.65-5.74)	Age, gender, BMI, waist-hip ratio, neck girth, and skinfolds thickness
Yusoff [31]	2010	289	1.87(1.02-3.4)*	Age, snoring, BMI, and neck circumference
Zou [32]	2012	344	Moderate OSA: 2.6(1.16-5.81)	Age, gender, BMI
O'Connor [33]	2009	2470	Mild OSA: 0.94(0.73-1.22)<br>Moderate OSA: 1.09(0.77-1.54)<br>Severe OSA: 1.5(0.91-2.46)	Age, gender, race, BMI, waist-hip ratio and neck girth.
Chan [34]	2016	587	1.50(1.05-2.15)*	Age, BMI, smoking

Abbreviations: OSA, obstructive sleep apnea; OR, odds ratio; 95%CI, 95% confidence interval; * the OR of the association between OSA and essential or resistant HTN compare to non-OSA participants
